# Adolescent intelligence and imaging-detected atherosclerosis in middle age: a population study of Swedish men

**DOI:** 10.1186/s12916-026-05140-z

**Published:** 2026-08-12

**Authors:** Hampus Eriksson, Ángel Herraiz-Adillo, Patrik Wennberg, Bledar Daka, Irene Esteban-Cornejo, Cecilia Lenander, Daniel Berglind, Ulrika Müssener, Fang Fang, Carl Johan Östgren, Karin Rådholm, Pontus Henriksson, Viktor H. Ahlqvist

**Affiliations:** 1 Department of Health, Medicine and Caring Sciences, Linköping University, Linköping, Sweden; 2 Department of Research in Kalmar, Region Kalmar County, Kalmar, Sweden; 3 Department of Public Health and Clinical Medicine, Umeå University, Umeå, Sweden; 4 Family medicine, School of public health and community medicine, Institute of medicine, Sahlgrenska Academy, Gothenburg University, Gothenburg, Sweden; 5 Department of Physical Education and Sports, Faculty of Sport Sciences, Sport and Health University Research Institute (iMUDS), University of Granada, Granada, 18071 Spain; 6 Instituto de Investigación Biosanitaria ibs.GRANADA, Granada, Spain; 7 Centro de Investigación Biomédica en Red Fisiopatología de la Obesidad y Nutrición (CIBERobn), Instituto de Salud Carlos III, Madrid, España; 8 Department of Clinical Sciences in Malmö, Centre for Primary Health Care Research, Lund University, Malmö, Sweden; 9 Department of Global Public Health Sciences, Karolinska Institutet, Stockholm, Sweden; 10 Centre for Epidemiology and Community Medicine, Stockholm County Council, Stockholm, Sweden; 11 Center for Wellbeing, Welfare and Happiness, Stockholm School of Economics, Stockholm, Sweden; 12 Institute of Environmental Medicine, Karolinska Institutet, Stockholm, Sweden; 13 Centre of Medical Image Science and Visualization (CMIV), Linköping University, Linköping, Sweden; 14 The George Institute for Global Health, University of New South Wales, Sydney, Australia; 15 Primary Health Care Center Kärna, Region Östergötland, Linköping, Sweden; 16 Department of Medical Sciences; Clinical Epidemiology, Uppsala University, Uppsala, Sweden; 17 Department of Biomedicine, Aarhus University, Aarhus, Denmark; 18 Department of Public Health and Caring Sciences; Clinical geriatrics, Uppsala University, Uppsala, Sweden

**Keywords:** Intelligence, Atherosclerosis, Coronary computed tomography, Coronary stenosis

## Abstract

**Background:**

Childhood or adolescent intelligence has been linked to major somatic diseases and premature mortality, but the underlying pathways remain unclear. The aim of this study was therefore to determine whether adolescent intelligence is associated with atherosclerosis in middle age and whether this association is mediated by the American Heart Association’s Life’s Essential 8 (LE8) metric of cardiovascular health markers.

**Methods:**

This population-based prospective cohort study linked data from men who underwent mandatory military conscription in adolescence with data from the Swedish CArdioPulmonary bioImage Study (SCAPIS). A standardized intelligence test was administered at conscription (mean age, 18.3 years; standard deviation [SD], 0.5), assessing logical reasoning, spatial ability, technical skills, and verbal comprehension. Scores were standardized to a mean of 100 (SD, 15). Atherosclerosis in middle age (mean age, 56.6 years; SD, 3.9) was assessed using coronary computed tomography angiography (CCTA) for coronary stenosis (0%, 1–49%, and ≥ 50%), coronary artery calcium (CAC) scores (0, 1–99, and ≥ 100 Agatston units), and carotid ultrasound for unilateral or bilateral carotid plaque/s. LE8 components assessed in SCAPIS were analyzed as potential mediators. Associations were modeled using multinomial logistic regression, and mediation was estimated using counterfactual mediation analysis.

**Results:**

A total of 8,117 men were included in the analysis of coronary stenosis, 7,958 for CAC, and 9,092 for carotid plaque. Higher adolescent intelligence was generally inversely associated with atherosclerosis in middle age. In analyses with adjustments (Model 2) for coronary stenosis, each 15-point (1 SD) increase in adolescent intelligence was associated with 17% lower odds of having ≥ 50% coronary stenosis (OR: 0.83; 95% CI: 0.75–0.90; P-value: <0.001). An intelligence difference between 70 and 130 corresponded to a prevalence difference of 4.1 percentage points (10.3% vs. 6.2%) of coronary stenosis ≥ 50%. Similar associations were observed for CAC scores and bilateral carotid plaques. Mediation via LE8 accounted for 41% to 68% of the association across outcomes.

**Conclusions:**

Higher adolescent intelligence was associated with a lower burden of atherosclerosis in middle age, an association that may be partly mediated by modifiable cardiovascular health factors.

**Supplementary Information:**

The online version contains supplementary material available at 10.1186/s12916-026-05140-z.

## Key Points

**Question:** Is there a relationship between adolescent intelligence and atherosclerosis in middle age, and could this association be mediated through cardiovascular health factors?

**Findings:** In this population-based cohort of Swedish men, higher adolescent intelligence was associated with a lower burden of atherosclerosis in middle age, measured using coronary computed tomography angiography and carotid ultrasound. About half of this association was mediated by modifiable cardiovascular health factors.

**Meaning:** These findings suggest that adolescent intelligence may shape cardiovascular health decades later, and this may be due to changes in cardiovascular health factors.

**Supplementary Information:**

The online version contains supplementary material available at 10.1186/s12916-026-05140-z.

## Background

 Higher childhood and adolescent intelligence have been associated with a lower risk of major cardiovascular (CVD), respiratory, and vascular-related neurodegenerative diseases later in life [1–3], as well as premature mortality [3–5]. These findings have been consistent across populations and study designs, including longitudinal cohort studies, genetic analyses, and family-based investigations [6–9].

However, few studies have examined how childhood or adolescent intelligence influences subclinical atherosclerosis later in life [10–13]. To the best of our knowledge, no previous study has used coronary computed tomography angiography (CCTA), an accurate bioimaging method that directly assesses coronary atherosclerosis. Unlike surrogate markers of atherosclerosis, CCTA can quantify coronary artery stenosis and characterize plaque phenotype, including calcified and non-calcified plaques. This may provide additional predictive value beyond established CVD risk prediction tools and surrogate atherosclerotic measures, such as coronary artery calcium (CAC) [14]. Some studies have examined intermediate risk factors as outcomes [15–18], while others have focused on diagnosed conditions or mortality [5, 19]. The former approach leaves uncertainty as to whether the findings extend to hard clinical endpoints, while the latter may be confounded by diagnostic bias (e.g., differential healthcare-seeking behavior) or non-traditional pathways to mortality (e.g., intelligence having an effect on accidental mortality but not on somatic disease mortality). As a result, it remains unclear whether differences in childhood or adolescent intelligence translate into behavioral patterns—such as smoking or dietary habits—that are substantial enough to influence long-term disease risk. Ideally, this question would be addressed in a unified cohort with childhood or adolescent intelligence data, midlife measures of behavioral health factors, and standardized assessments of subclinical disease across all participants.

To address this gap, we linked historical military conscription records with contemporary data from the Swedish CArdioPulmonary bioImage Study (SCAPIS). Using CCTA to evaluate coronary stenosis, CAC scoring, and carotid ultrasound to detect plaque, we examined whether adolescent intelligence is associated with atherosclerosis in middle age, and to what extent this relationship is mediated by traditional cardiovascular health factors.

## Aim

To determine whether adolescent intelligence is associated with atherosclerosis in middle age and whether this association is mediated by the American Heart Association’s Life’s Essential 8 (LE8) metric of cardiovascular health.

## Methods

### Study design

A longitudinal study was constructed by linking male participants from SCAPIS to their historical conscription records in the Swedish Military Conscription Register (SMCR). Detailed information about SCAPIS and SMCR has been published elsewhere [20, 21]. In brief, SMCR contains data on almost all Swedish men who underwent military conscription assessments around the age of 18 years. Military conscription was compulsory during the period when participants reached adolescence. Participants underwent conscription between 1972 and 1987, and SMCR data were linked to SCAPIS using personal identification numbers.

SCAPIS is a population-based study of 50–64-year-olds conducted at six university hospitals across Sweden (Linköping, Gothenburg, Stockholm, Umeå, Uppsala, and Malmö/Lund), comprising 30,154 participants (men = 14,646) who underwent comprehensive state-of-the-art bioimaging and cardiovascular health assessments. Participants also completed detailed questionnaires on health, lifestyle, and sociodemographic factors (Additional file 1: Table S1) [20–31]. The collection period was between 2013 and 2018.

### Derivation of analytical sample

Inclusion criteria were men who had participated in SCAPIS and had SMCR data from conscription before 20 years of age (Additional file 1: Fig. S1). For the primary complete case analysis, we excluded individuals with missing data on adolescent intelligence at conscription, or on the covariates BMI at conscription, educational level at conscription, physical fitness at conscription, age at conscription, site of conscription, year of conscription, and SCAPIS site. To maximize the number of participants included, we performed outcome-specific analyses, excluding participants with missing data separately for each outcome. Thus, participants with missing data on CCTA, CAC, or carotid plaque were excluded only from the corresponding analysis. This resulted in sample sizes of 8,117 for CCTA, 7,958 for CAC, and 9,092 for carotid plaque (Additional file 1: Fig. S1).

### Adolescent intelligence

The young men (< 20 years) underwent a series of cognitive and physical assessments during conscription to evaluate their suitability for military service and appropriate role placement [20, 32]. The cognitive assessment comprised four standardized paper-and-pen tests evaluating: logical reasoning (ability to process instructions and make reasoned decisions), spatial ability (‘visualization’ – manipulation of objects in mind and ‘spatial relations’ – the ability to see and recognize still objects in different positions), technical skills (existing technical ability – used to assess suitability for technical and mechanical positions), and verbal comprehension (linguistic understanding and ability to use oral and written language). Each test consisted of 40 items and jointly meant to provide an extensive battery to match the concept of general ability, inspired by contemporary intelligence tests.

The scores from these tests were summed (unweighted) to produce a total cognitive score, which was then standardized to a normal distribution within each conscription year to account for temporal differences and enable comparisons across conscription waves. To enhance comparability with other studies, we centered the distribution to a mean of 100 with a standard deviation (SD) of 15. While the exact content of the sub-tests remains confidential due to military regulations, they have been widely utilized in cognitive research and have demonstrated strong construct validity in measuring latent general intelligence [32, 33]. Additionally, they have been shown to correlate strongly with results on the Swedish Scholastic Aptitude Test [34].

### Atherosclerosis in middle age

Three different types of categorical outcomes were used to evaluate the grade of atherosclerosis: coronary stenosis, CAC score, and carotid plaque [21]. First, CCTA, a highly accurate bioimaging technique, was used to assess the grade of stenosis by considering the segment with the highest level of stenosis in any of the 11 most relevant coronary artery segments (1–3, 5–7, 9, 11–13, and 17) according to Society of Cardiovascular Computed Tomography guidelines [26, 35]. For CCTA, imaging was conducted using a computed tomography (CT) with a Stellar Detector and dual-source (Siemens, Forchheim, Germany). Coronary stenosis was graded as follows: 0%, 1–49% or ≥ 50% stenosis since ≥ 50% stenosis in the proximal segment increased the prognostic value of mortality during 2.3 years of follow-up [36]. Calcium blooming artifact on the CCTA was classified as 1–49% stenosis, while the presence of a coronary stent was classified as ≥ 50% stenosis. Second, CT was used to generate a CAC score based on the calcium levels in the coronary arteries [27, 37]. An international standard protocol was used to evaluate the CAC score [27]. CT imaging for CAC was also performed using a Stellar Detector and dual-source (Siemens, Forchheim, Germany). The scores were categorized as follows: 0, 1–99 or ≥ 100 Agatston units. For CCTA stenosis and CAC, the analyses were restricted to participants having data in all of the four proximal coronary segments (1, 5, 6, and 11). The prognostic value of CCTA and CAC in asymptomatic individuals supports their validity as imaging markers of coronary atherosclerosis [35].

Third, ultrasound on carotid arteries was used to evaluate the carotid plaque/s. A Siemens Acuson S2000 (Siemens, Forchheim, Germany) with two-dimensional greyscale and a linear 9L4 transducer (Siemens, Forchheim, Germany) was used as equipment. The carotid plaque was defined according to the Mannheim consensus, exceeding > 50% of the adjacent intima-media thickness, extending > 0.5 mm into the lumen or an overall plaque thickness > 1.5 mm [28]. The categorization for the plaque was divided as follows: no plaque, unilateral plaque/s and bilateral plaques.

### Cardiovascular health behaviors and factors in middle age

To examine how cardiovascular health behaviors and factors mediate the association between adolescent intelligence and middle age atherosclerosis, we applied the American Heart Association’s LE8 framework. This composite index includes eight health components: four health behaviors (physical activity, diet, sleep health, and smoking habits) and four health factors (blood pressure [BP], BMI, blood lipids, and blood glucose). Use of medication for hypertension, blood lipids, and blood glucose was also considered. The LE8 score ranges from 0 to 100 (ideal cardiovascular health). All components were assessed at the time of SCAPIS participation [31, 36]. Therefore, data for the outcomes and mediating variables were collected concurrently. Details of the LE8 scoring and each component are presented (Additional file 1: Table S2) [22–25, 29–31].

### Covariates

A directed acyclic graph was constructed to identify potential confounding (Additional file 1: Fig. S2). Covariates included: BMI measured at conscription using calibrated scales and stadiometers (kg/m²), and physical fitness at conscription assessed via knee extension strength (N) [37] and cardiorespiratory fitness from a maximal cycle ergometer test (W). Educational level (attained or enrolled) at conscription was classified as primary school, secondary school, university-level education or unknown. To account for secular trends, year of conscription was grouped into eight two-year intervals spanning 1972–1987. Additional covariates included sites at conscription, age at conscription, age at SCAPIS participation, and site at SCAPIS. Continuous covariates were modeled using both linear and cubic terms to account for potentially non-linear associations.

### Statistical analysis

Descriptive statistics are reported as means with SD or counts with percentages. To facilitate analysis and capture variability in the outcomes, a three-level categorical outcome was analyzed using multinomial logistic regression, with no atherosclerosis as the reference category. Models were used to estimate covariate-adjusted odds ratio (OR), prevalences (100 × marginal probability), and 95% confidence interval (CI) for each outcome category. Intelligence was modeled both linearly—per 15 units (equivalent to 1 SD, consistent with prior literature)—and using restricted cubic splines with knots at the 5th, 35th, 65th, and 95th percentiles [38]. To model the adjusted prevalences of coronary stenosis across each of the 11 most clinically relevant coronary segments, we employed a random-effects multinomial logistic regression, allowing the outcome in each segment to correlate within the individual.

Finally, to explore potential mechanisms, we conducted counterfactual mediation analyses to decompose the total effect of adolescent intelligence on atherosclerosis in middle age into direct and indirect (mediated) components, along with the mediated proportion through cardiovascular health: i.e., LE8 total score, LE8 factors score, LE8 behaviors score and each LE8 component [36]. The mediation analyses were performed using subsamples of all the participants with complete data for the relevant mediator variables at SCAPIS. In addition, mediation analyses were conducted considering socioeconomic indicators at SCAPIS including three different analyses with educational level, and financial strain as potential mediators. Financial strain was assessed using two questions: (1) having serious financial problems and (2) having difficulties managing regular expenses during the previous 12 months.

Two covariate adjustment models were specified to separate different levels of adjustments. Model 1 included age at conscription, site at conscription, year of conscription, site at SCAPIS, and age at SCAPIS. Model 2 built on Model 1 by additionally adjusting for BMI at conscription, physical fitness (cardiorespiratory fitness and muscle strength) at conscription, and educational level measured at conscription. Results from Model 1 are mostly presented in the Additional file 1. All analyses were conducted using Stata versions 18 and 19.

### Sensitivity analyses

We performed several sensitivity analyses to assess the robustness of our findings. First, we conducted additional analyses with adjustment for: (1) self-reported smoking status at conscription (derived retrospectively from SCAPIS data), systolic and diastolic BP at conscription, and height at conscription, the latter serving as a marker of secular changes in growth and health conditions (i.e., the Flynn effect [39] ); (2) educational level at SCAPIS; and (3) socioeconomic indicators at SCAPIS, including educational level, and questions about having serious financial problems and having difficulties managing regular expenses during the previous 12 months. Although we hypothesized that socioeconomic indicators at SCAPIS (i.e., midlife) would primarily act as a mediator, we also explored its potential role as a confounder, as these midlife measures may partly proxy socioeconomic circumstances at conscription that could have confounded the association. Similarly, although we considered lipid-lowering medication to be used primarily as a potential mediator, we additionally adjusted for it in a sensitivity analysis to assess the robustness of the results.

In addition, because many participants had unknown educational level, we performed a sensitivity analysis without adjusting for educational level or BMI at conscription. We also performed additional analyses excluding participants with: (1) unknown educational level at conscription; and (2) CVD at SCAPIS, including myocardial infarction, stroke, peripheral artery disease, and coronary artery bypass graft surgery. Finally, sensitivity analyses using alternative categorisation of atherosclerosis in middle age were conducted by: (1) classifying calcium blooming artifacts as indicating ≥ 50% coronary stenosis rather than 1–49% stenosis; (2) excluding participants with coronary artery stents instead of categorizing them as having ≥ 50% stenosis; and (3) restricting the analysis to participants with complete data for all 11 coronary segments.

## Results

### Descriptive characteristics

There were 8,117, 7,958 and 9,092 participants available for the analyses in coronary stenosis, CAC and carotid plaque, respectively. At conscription, the 8,117 adolescents with coronary stenosis data had a mean (SD) age of 18.3 (0.5) years, an intelligence score of 100.5 (14.7) points, a BMI of 21.2 (2.3) kg/m^2^, a knee strength of 556.9 (113.2) N, and a cardiorespiratory fitness of 258.8 (42.0) W. Educational level at conscription was distributed as primary (17.9%), secondary (64.7%), university (0.3%), and unknown (17.2%) (Table 1; Additional file 1: Tables S3-S4). Descriptive statistics for participants with known compared with unknown educational level at conscription are presented (Additional file 1: Table S5).

**Table 1:** Descriptive characteristics of the participants included in the analyses of coronary stenosis, coronary artery calcium, and carotid plaque

Characteristics	Coronary stenosis	CAC	Carotid plaque
	8,117 (100.0%)	7 ,958 (100.0%)	9,092 (100.0%)
**At conscription (baseline)**			
Adolescent intelligence	100.5 (14.7)	100.5 (14.7)	100.3 (14.8)
Age (years)	18.3 (0.5)	18.3 (0.5)	18.3 (0.5)
Height (cm)	179.8 (6.4)	179.8 (6.4)	179.7 (6.5)
Weight (kg)	68.7 (8.9)	68.8 (8.9)	68.9 (9.0)
BMI (kg/m^2^)	21.2 (2.3)	21.3 (2.3)	21.3 (2.4)
Smoking status			
Current smoker	2,167 (26.7%)	2,111 (26.5%)	2,453 (27.0%)
Non-smoker	5,694 (70.1%)	5,597 (70.3%)	6,324 (69.6%)
Unknown	256 (3.2%)	250 (3.1%)	315 (3.5%)
Systolic BP (mmHg)	127.8 (10.6)	127.8 (10.6)	127.9 (10.7)
Diastolic BP (mmHg)	67.9 (9.5)	67.9 (9.5)	68.0 (9.5)
Cardiorespiratory fitness (W)	258.8 (42.0)	258.9 (42.0)	258.3 (41.9)
Knee strength (N)	556.9 (113.2)	557.0 (113.3)	556.6 (113.7)
Education level			
Primary school	1,454 (17.9%)	1,419 (17.8%)	1,652 (18.2%)
Secondary school	5,248 (64.7%)	5,151 (64.7%)	5,855 (64.4%)
University degree	22 (0.3%)	22 (0.3%)	29 (0.3%)
Unknown	1,393 (17.2%)	1,366 (17.2%)	1,556 (17.1%)
**At SCAPIS (follow-up)**			
Follow-up (years)	38.3 (3.7)	38.3 (3.7)	38.3 (3.8)
Age (years)	56.6 (3.9)	56.6 (3.9)	56.6 (3.9)
Previous CVD			
No	7,857 (96.8%)	7,782 (97.8%)	8,720 (95.9%)
Yes	260 (3.2%)	176 (2.2%)	372 (4.1%)

Continuous variables are presented as mean (SD), and categorical variables as n (%). CVD was defined as myocardial infarction, stroke, peripheral artery disease, or coronary artery bypass surgery.

Abbreviations: *BMI * body mass index,* BP *blood pressure, *CAC *coronary artery calcium,* CVD* cardiovascular disease,* N *newton, *SCAPIS *Swedish CArdioPulmonary bioImage Study, *SD* standard deviation, *W* watt

At SCAPIS recruitment, mean age of participants was 56.6 (3.9) years. At that time, 3,872 (47.7%) participants presented with 0% coronary stenosis, 3,590 (44.2%) with 1–49% coronary stenosis and 655 (8.1%) with ≥ 50% coronary stenosis. According to CAC, 3,960 (49.8%) had 0 Agatston units, 2,747 (34.5%) had 1–99 Agatston units and 1,251 (15.7%) had ≥ 100 Agatston units. Finally, 3,714 (40.8%) had no plaque, 2,789 (30.7%) had unilateral plaque/s and 2,589 (28.5%) had bilateral plaques (Table 2).

### Adolescent intelligence and atherosclerosis

After adjusting for covariates, higher adolescent intelligence was inversely associated with measures of atherosclerosis in middle age (Table 2). In Model 2, each 15-point (1 SD) higher intelligence was associated with 9% lower odds of having 1–49% coronary stenosis (OR: 0.91; 95% CI: 0.87–0.96), and 17% lower odds of having ≥ 50% coronary stenosis (OR: 0.83; 95% CI: 0.75–0.90). Similar associations were observed for the other two outcomes: each 15-point (1 SD) higher intelligence was linked to 10% lower odds of having a CAC score ≥ 100 Agatston units (OR: 0.90; 95% CI: 0.83–0.96) and 12% lower odds of bilateral carotid plaques (OR: 0.88; 95% CI: 0.83–0.93).

**Table 2:** Association of adolescent intelligence (per 15-unit [1 SD] increase) with coronary stenosis, coronary artery calcium, and carotid plaques in middle age, estimated using multinomial logistic regression

Outcomes	Number of participants	Per 15-unit (1 SD) higher adolescent intelligence					
		Model 1			Model 2		
		OR	95% CI	P	OR	95% CI	P
Coronary stenosis	8,117 (100.0%)						
* 0% stenosis*	3,872 (47.7%)	1.00	–	–	1.00	–	–
* 1–49% stenosis*	3,590 (44.2%)	0.90	0.86–0.94	< 0.001	0.91	0.87–0.96	< 0.001
* ≥ 50% stenosis*	655 (8.1%)	0.79	0.72–0.86	< 0.001	0.83	0.75–0.90	< 0.001
Coronary artery calcium	7,958 (100.0%)						
* 0 Agatston units*	3,960 (49.8%)	1.00	–	–	1.00	–	–
* 1–99 Agatston units*	2,747 (34.5%)	0.91	0.87–0.96	< 0.001	0.93	0.88–0.98	0.008
* ≥ 100 Agatston units*	1,251 (15.7%)	0.87	0.81–0.93	< 0.001	0.90	0.83–0.96	0.003
Carotid plaque	9,092 (100.0%)						
* No plaque*	3,714 (40.8%)	1.00	–	–	1.00	–	–
* Unilateral plaque/s*	2,789 (30.7%)	0.97	0.92–1.02	0.218	0.96	0.91–1.01	0.140
* Bilateral plaques*	2,589 (28.5%)	0.87	0.82–0.91	< 0.001	0.88	0.83–0.93	< 0.001

Model 1 adjusts for age at conscription, site at conscription, year at conscription, site at SCAPIS, age at SCAPIS

Model 2 builds on Model 1 by additionally adjusting for BMI at conscription, educational level at conscription and physical fitness at conscription

Abbreviations: *BMI* body mass index, *CI* confidence interval, *OR* odds ratio, *SCAPIS* Swedish CArdioPulmonary bioImage Study, *SD* standard deviation

**Fig. 1:**
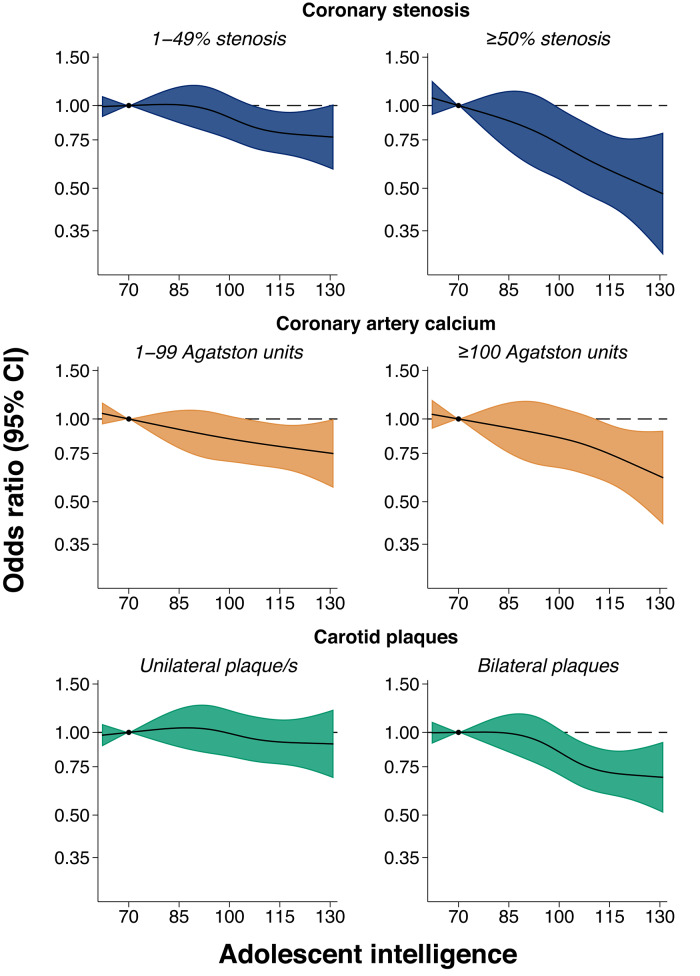
Association between adolescent intelligence and atherosclerosis in middle age. Odds ratios and 95% CI are shown for coronary stenosis, coronary artery calcium, and carotid plaque/s, modeled using multinomial logistic regression with no atherosclerosis as the reference category and restricted cubic splines. The reference value (dot) is an adolescent intelligence score of 70, corresponding to 2 SD below the mean. Estimates are from Model 2, adjusted for age at conscription, site at conscription, year at conscription, site at SCAPIS, age at SCAPIS, BMI at conscription, educational level at conscription and physical fitness at conscription. Abbreviations: BMI, body mass index; CI, confidence interval; OR, odds ratio; SCAPIS, Swedish CArdioPulmonary bioImage Study

Findings were consistent when modeling adolescent intelligence as a non-linear exposure using restricted cubic splines (Fig. 1; Additional file 1: Fig. S3). In the adjusted Model 2, results for both coronary stenosis and CAC demonstrated a graded, inverse association, with higher adolescent intelligence linked to progressively lower levels of atherosclerosis in middle age. The inverse association appeared more pronounced for severe outcomes, particularly for coronary stenosis ≥ 50% and CAC ≥ 100 Agatston units. Associations with carotid plaque/s appeared generally weaker than those observed for coronary measures.

### Absolute risk across the 11 most relevant coronary artery segments

**Fig. 2:**
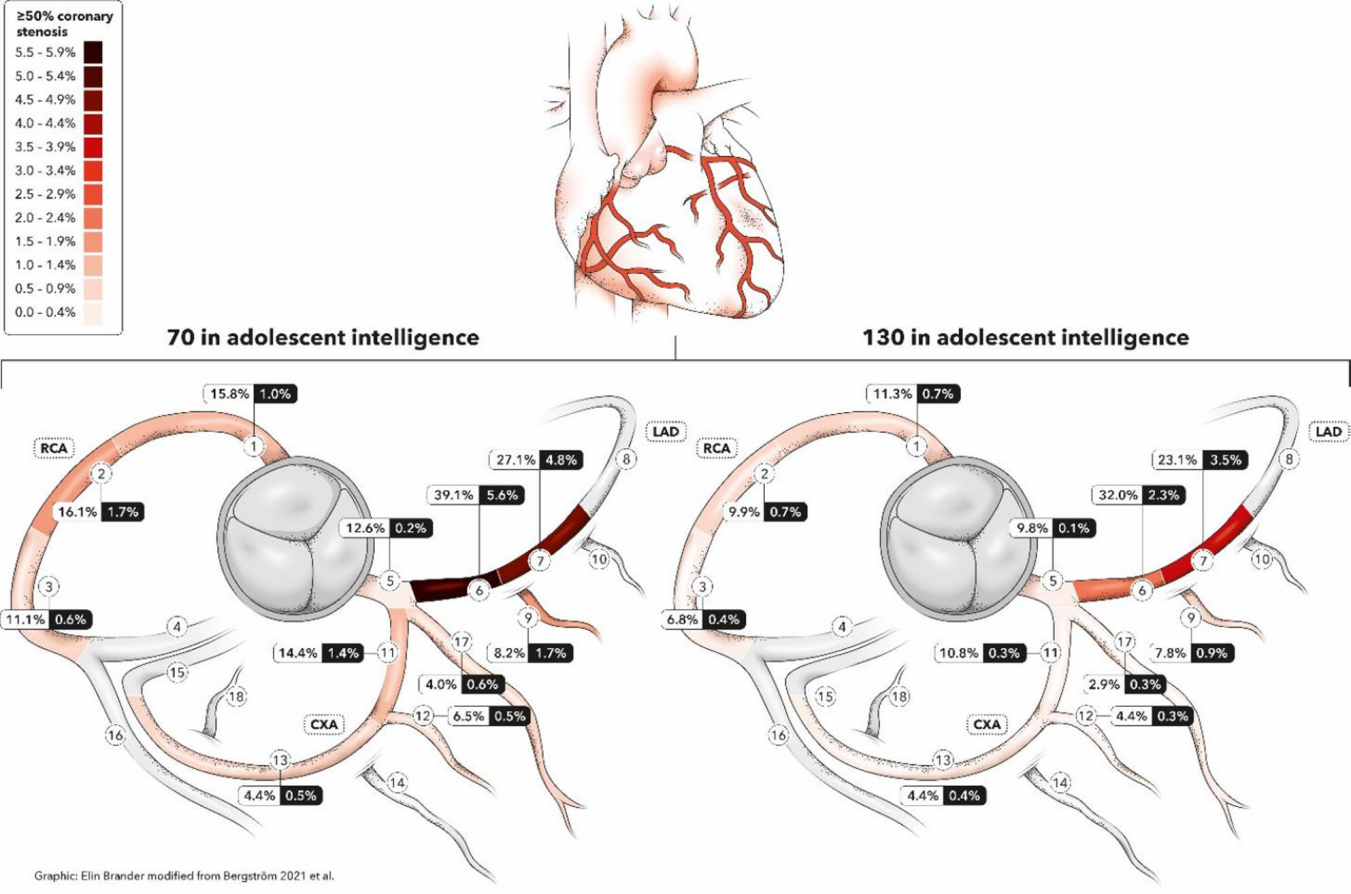
Adjusted prevalences of coronary stenosis detected by CCTA across the 11 most relevant coronary artery segments in middle age, stratified by adolescent intelligence (70 vs. 130). Estimates are from Model 2, which is adjusted for age at conscription, site at conscription, year at conscription, site at SCAPIS, age at SCAPIS, BMI at conscription, educational level at conscription and physical fitness at conscription. Coronary artery segments are defined according to the Society of Cardiovascular Computed Tomography guidelines [26]. White and black boxes show adjusted prevalences of coronary stenosis 1–49% and ≥ 50%, respectively. Abbreviations: CCTA, coronary computed tomography angiography; CXA, Circumflex coronary artery; LAD, Left anterior descending coronary artery; RCA, Right coronary artery; SCAPIS, Swedish CArdioPulmonary bioImage Study

In general, individuals with lower adolescent intelligence had a higher adjusted prevalence of coronary atherosclerosis across segments compared to those with higher intelligence (Fig. 2; Additional file 1: Tables S6-S7).

Overall, an intelligence difference between 70 and 130 corresponded to a 5.7 percentage point difference in the prevalence of 1–49% coronary stenosis (47.0% vs. 41.3%) and a 4.1 percentage point difference in the prevalence of ≥ 50% stenosis (10.3% vs. 6.2%).

### Mediation through traditional cardiovascular health factors

**Fig. 3:**
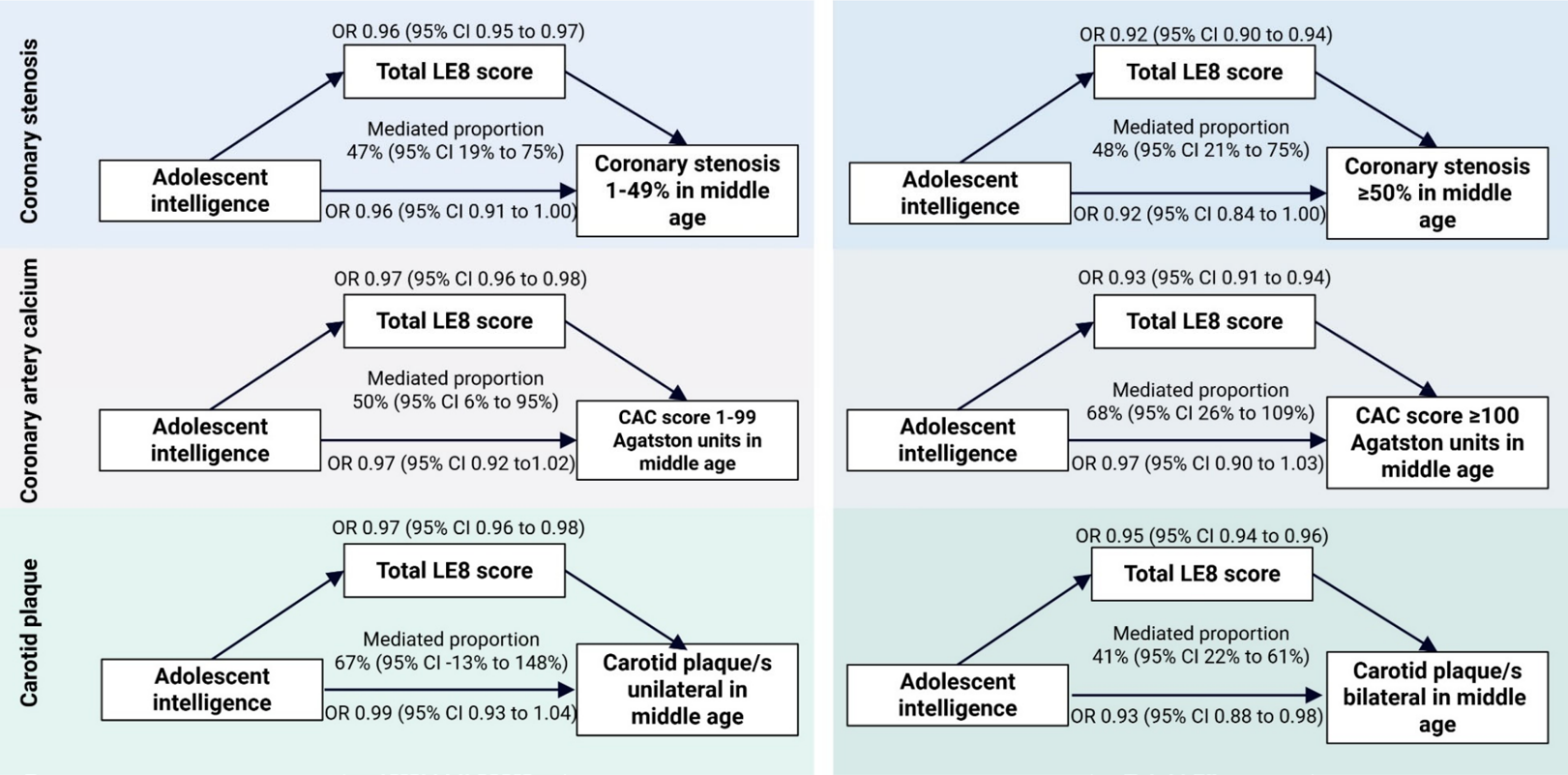
Direct and indirect effects of adolescent intelligence on coronary stenosis, coronary artery calcium, and carotid plaques in middle age, mediated via Life’s Essential 8 from SCAPIS, estimated using counterfactual mediation analysis. The LE8 variable included physical activity, diet, sleep health, and smoking habits, blood pressure, BMI, blood lipids, and blood glucose from SCAPIS. Mediated proportions are presented as percentages (note this estimate and its 95% CI is not bounded between 0 and 100). Estimates are from Model 2, which is adjusted for age at conscription, site at conscription, year at conscription, site at SCAPIS, age at SCAPIS, BMI at conscription, educational level at conscription and physical fitness at conscription. Sample sizes were 7,905 participants for coronary stenosis, 7,752 participants for CAC and 8,820 participants for carotid plaque. Abbreviations: BMI, Body Mass Index; CAC, coronary artery calcium; CI, confidence interval; LE8, Life’s Essential 8; OR, odds ratio; SCAPIS, Swedish CArdioPulmonary bioImage Study

The percentage of the association mediated through the total LE8 score measured at SCAPIS was 48% for coronary stenosis (≥ 50%), 68% for CAC (≥ 100 Agatston units), and 41% for bilateral carotid plaques (Fig. 3; Additional file 1: Table S8). The mediated proportion was somewhat larger for most LE8 factors than for LE8 behaviors (Additional file 1: Table S8), with the highest contributions being observed for BP, blood lipids, and BMI for coronary stenosis (≥ 50%) (Additional file 1: Tables S9-S11).

The mediated proportion from socioeconomic indicators ranged from 0% to 50%, with the highest contributions observed for educational level at SCAPIS in carotid plaque outcomes (Additional file 1: Table S12).

### Sensitivity analyses

Results were similar to those in the main analyses after extended adjustments for (1) smoking status, systolic and diastolic BP, and height at conscription (Additional file 1: Table S13); (2) educational level at SCAPIS (Additional file 1: Table S14); (3) socioeconomic indicators at SCAPIS (Additional file 1: Table S14); or (4) lipid-lowering medication at SCAPIS (Additional file 1: Table S15). Results were also similar when we (5) did not adjust for educational level at conscription (Additional file 1: Table S16); (6) did not adjust for BMI at conscription (Additional file 1: Table S17); (7) excluded participants with unknown educational level at conscription (Additional file 1: Table S18); or (8) excluded participants with CVD at SCAPIS (Additional file 1: Table S19). Finally, results were similar when we (9) classified calcium blooming artifacts as indicating ≥ 50% coronary stenosis (Additional file 1: Table S20); (10) excluded participants with coronary artery stents (Additional file 1: Table S20); or (11) restricted the analysis to participants with complete data for all 11 coronary segments (Additional file 1: Table S21).

## Discussion

In this observational population-based cohort study of Swedish men, with nearly four decades of follow-up, higher adolescent intelligence was dose-dependently associated with a lower burden of imaging-detected atherosclerosis in middle age. For instance, each 15-unit increase (1 SD) in adolescent intelligence was linked to 17% lower odds of coronary stenosis (≥ 50%). Some analyses showed that the associations were more pronounced for coronary stenosis and CAC than for carotid plaque. The weaker association with carotid plaque is consistent with another longitudinal study examining adolescent BP in relation to atherosclerosis in middle age [40]. While true biological differences in the association between intelligence and different arterial territories may exist, we cannot exclude the possibility that differences in the diagnostic performance of the methods used to assess atherosclerosis also contributed to these findings.

The adjusted prevalences for coronary stenosis (≥ 50%) were 10.3% in participants with 70 in adolescent intelligence compared with 6.2% in participants with 130 in adolescent intelligence. Finally, a substantial proportion of the association between adolescent intelligence and middle age atherosclerosis was mediated by cardiovascular health (measured as total LE8 score), particularly cardiovascular health factors.

### Comparisons with existing literature

To our knowledge, only four prior studies have investigated the link between childhood or adolescent intelligence and subclinical markers of CVD later in life [10–13]. In another Swedish study of 1,009 men, lower scores on conscription adolescent intelligence tests were associated with increased risk of carotid plaques, an association that appeared to be mediated by lifestyle habits and socioeconomic status [11]. A U.S study of 4,286 male veterans found that lower intelligence in late adolescence was associated with a lower ankle–brachial index (ABI) later in life [10]. Another study, based on a smaller UK cohort (*n* = 412), reported that higher intelligence at age 11 was associated with lower carotid intima–media thickness in adulthood [12]. However, a separate UK study with longer follow-up found no differences when studying the childhood intelligence measures and carotid intima-media thickness, carotid stenosis > 25%, or ABI < 0.9 at age 73 [13]. While informative, these prior studies were limited by relatively small sample sizes and reliance on indirect or surrogate markers of vascular health. In contrast, CCTA provides high-resolution, direct visualization of coronary stenosis and plaque phenotype—including calcified, non-calcified, and mixed plaques. Our findings therefore extend these prior works by offering a more comprehensive, imaging-detected assessment of atherosclerosis. In addition, this study demonstrates that cardiovascular health factors mediate the association between adolescent intelligence and atherosclerosis in middle age.

### Strength and limitations

Our study has several notable strengths. First, we used high-resolution CCTA, which allows precise quantification of coronary stenosis and characterization of both calcified and non-calcified plaques. In contrast, CAC is an indirect marker of coronary atherosclerosis because it quantifies coronary calcium but does not directly assess the degree of coronary stenosis. Both CAC and CCTA have been shown to improve prediction of all-cause mortality in asymptomatic patients, supporting their validity as imaging-detected measures of coronary atherosclerosis [35]. Accordingly, CCTA provides more detailed information on coronary stenosis and improves prediction of coronary events compared with CAC [14].

Second, the nearly 40-year follow-up provides a rare opportunity to examine long-term associations from adolescence into late middle age. Third, the study includes a large, well-characterized cohort, addressing the limitations of small sample sizes that have affected prior research in this area [10, 12, 13, 41].

Several limitations should also be considered. First, women were not represented in the dataset, as the study population comprised only men at military conscription, which limits the generalizability of the findings to female populations. Albeit we might speculate that any potential mechanism may be similar in women, it should be noted that men tend to develop atherosclerosis and cardiovascular events earlier, partly due to less favorable cardiovascular risk profiles [42, 43]. Corresponding studies in women are needed to determine whether similar associations and mediating pathways are present in female populations. Second, conscription procedures also excluded individuals with significant functional disabilities (e.g., intellectual disability). Accordingly, the findings may not generalize to these groups and should be interpreted as reflecting associations within the broader general population rather than pertaining to individuals with functional disabilities. Third, as with any observational study, there remains a risk of residual confounding due to unmeasured social, behavioral, or biological factors. Future studies should leverage complementary designs with orthogonal bias structures, such as instrumental variable approaches, negative control analyses, and sibling-comparison designs, to further interrogate the role of early-life cognitive ability in long-term atherosclerosis risk. Fourth, although long follow-up is a strength, the lack of repeated imaging on atherosclerosis over time limits our ability to pinpoint when differences in atherosclerosis emerge. Fifth, because LE8 and the outcomes were assessed concurrently, the temporal ordering of the mediation pathway cannot be established. Reverse causation is possible, particularly for behavioral components, and the mediation analyses should therefore be interpreted as exploratory rather than causal. Future studies incorporating repeated assessments of cardiovascular health profiles across the life course would help clarify these pathways.

### Clinical implications

To the best of our knowledge, this is the first study to investigate the relationship between adolescent intelligence and CCTA, an accurate bioimaging method for assessing atherosclerosis in middle age, both according to relative and absolute metrics. Our study provides evidence of an association between lower adolescent intelligence and increased risk of imaging-detected atherosclerosis and explores potential pathways linking cognitive ability in adolescence to later-life health outcomes.

As this is an observational study, causal inference is limited. Nevertheless, the magnitude of the observed association was generally comparable to that reported for some established cardiovascular risk factors. For instance, each 15-point higher adolescent intelligence was associated with a 17% lower odds of coronary stenosis ≥ 50% (OR: 0.83; 95% CI: 0.75–0.90), which is roughly comparable to the 22% lower odds observed in adolescents in the top third of cardiorespiratory fitness compared with those in the bottom third (OR: 0.78; 95% CI: 0.61–0.99) [44].

The adjusted prevalences highlight the potential impact of adolescent intelligence: for coronary stenosis (≥ 50%), having an adolescent intelligence score of 70 versus 130 was associated with a 4.1-percentage point higher marginal prevalence, compared with a 2.6-percentage point difference for low cardiorespiratory fitness and low strength versus high cardiorespiratory fitness and high strength in the same cohort [44].

## Conclusions

In this large observational population-based study of Swedish men, higher adolescent intelligence was associated with a lower burden of atherosclerosis in middle age, including coronary stenosis, CAC, and carotid plaque. This association may be mediated by modifiable cardiovascular health factors.

## Supplementary Information

Below is the link to the electronic supplementary material.

Supplementary Material 1: Additional File 1: Tables S1–S21, Figures S1–S3.

## Data Availability

The data used for the applied example are publicly unavailable according to regulations under Swedish law. Readers interested in obtaining the data may seek similar approvals and inquire through https://www.scapis.org/.

## Abbreviations

ABI: ankle–brachial index
BMI: body mass index
BP: blood pressure
CAC: coronary artery calcium
CCTA: coronary computed tomography angiography
CI: confidence interval
CT: computed tomography
CVD: cardiovascular disease
LE8: Life’s Essential 8
OR: odds ratio
SCAPIS: Swedish CArdioPulmonary bioImage Study
SD: standard deviation
SMCR: Swedish Military Conscription Register

## References

[1] Longinetti E, Zhan Y, Sata M, Larsson H, D′Onofrio BM, Iso H, et al. Heart rate, intelligence in adolescence, and Parkinson’s disease later in life. Eur J Epidemiol. 2021;36:1055–64. 33675447 10.1007/s10654-021-00730-y PMC8542538

[2] Deary IJ, Hill WD, Gale CR. Intelligence, health and death. Nat Hum Behav. 2021;5:416–30. 33795857 10.1038/s41562-021-01078-9

[3] Christensen GT, Mortensen EL, Christensen K, Osler M. Intelligence in young adulthood and cause-specific mortality in the Danish Conscription Database – A cohort study of 728,160 men. Intelligence. 2016;59:64–71.

[4] Twig G, Tirosh A, Derazne E, Haklai Z, Goldberger N, Afek A, et al. Cognitive function in adolescence and the risk for premature diabetes and cardiovascular mortality in adulthood. Cardiovasc Diabetol. 2018;17:154. 30518353 10.1186/s12933-018-0798-5 PMC6280532

[5] Sörberg Wallin A, Allebeck P, Gustafsson JE, Hemmingsson T. Childhood IQ and mortality during 53 years’ follow-up of Swedish men and women. J Epidemiol Community Health (1978). 2018;72:926–32. 10.1136/jech-2018-210675 29925669

[6] Calvin CM, Deary IJ, Fenton C, Roberts BA, Der G, Leckenby N, et al. Intelligence in youth and all-cause-mortality: systematic review with meta-analysis. Int J Epidemiol. 2011;40:626–44. 21037248 10.1093/ije/dyq190 PMC3147066

[7] Davies NM, Hill WD, Anderson EL, Sanderson E, Deary IJ, Smith GD. Multivariable two-sample Mendelian randomization estimates of the effects of intelligence and education on health. Elife. 2019;8:e43990. 31526476 10.7554/eLife.43990 PMC6748790

[8] Yang F, Hu T, Chen S, Wang K, Qu Z, Cui H. Low Intelligence Predicts Higher Risks of Coronary Artery Disease and Myocardial Infarction: Evidence From Mendelian Randomization Study. Front Genet. 2022;13:756901. 35198002 10.3389/fgene.2022.756901 PMC8859249

[9] Jokela M, Batty GD, Deary IJ, Silventoinen K, Kivimäki M. Sibling Analysis of Adolescent Intelligence and Chronic Diseases in Older Adulthood. Ann Epidemiol. 2011;21:489–96. 21440456 10.1016/j.annepidem.2011.01.008

[10] Gale CR, Deary IJ, Fowkes RFG, Batty GD. Intelligence in early adulthood and subclinical atherosclerosis in middle-aged men: the Vietnam Experience Study. J Epidemiol Community Health (1978). 2012;66:e13. 10.1136/jech.2010.109835 21558481

[11] Norberg M, Liv P, Näslund U, Wester P, Andersson EM, Nordin S. The Path for Men from Young Adulthood Results of Cognitive Tests to Subclinical Atherosclerosis at Age 60: The Mediating Role of Socioeconomic Status, Lifestyle and Cardiovascular Disease Risk Factors–Results from a VIPVIZA Study. Rev Cardiovasc Med. 2025;26:26312. 40160597 10.31083/RCM26312 PMC11951286

[12] Roberts BA, Batty GD, Gale CR, Deary IJ, Parker L, Pearce MS. IQ in childhood and atherosclerosis in middle-age: 40 Year follow-up of the Newcastle Thousand Families Cohort Study. Atherosclerosis. 2013;231:234–7. 24267233 10.1016/j.atherosclerosis.2013.09.018 PMC3918147

[13] Gale CR, Eadie E, Thomas A, Bastin ME, Starr JM, Wardlaw J, et al. Intelligence in Childhood and Atherosclerosis of the Carotid and Peripheral Arteries in Later Life: The Lothian Birth Cohort 1936. PLoS ONE. 2015;10:e0125280. 25915652 10.1371/journal.pone.0125280 PMC4411126

[14] Bergström G, Engström G, Björnson E, Adiels M, Andersson JSO, Andersson T, et al. Coronary Computed Tomography Angiography in Prediction of First Coronary Events. JAMA. 2026;335:245–54. 41206900 10.1001/jama.2025.21077 PMC12598578

[15] Kumpulainen SM, Heinonen K, Pesonen AK, Salonen MK, Andersson S, Lano A, et al. Childhood cognitive ability and physical activity in young adulthood. Health Psychol. 2017;36:587–97. 28541087 10.1037/hea0000493

[16] Altschul DM, Starr JM, Deary IJ. Cognitive function in early and later life is associated with blood glucose in older individuals: analysis of the Lothian Birth Cohort of 1936. Diabetologia. 2018;61:1946–55. 29860628 10.1007/s00125-018-4645-8 PMC6096629

[17] Druffner N, Egan D, Ramamurthy S, O’Brien J, Davis AF, Jack J, et al. IQ in high school as a predictor of midlife alcohol drinking patterns. Alcohol Alcohol. 2024;59:agae035. 38804536 10.1093/alcalc/agae035

[18] Wimmelmann CL, Grønkjær M, Mortensen EL. Changes in BMI from young adulthood to late midlife in 1536 Danish men: The influence of intelligence and education. Obes Med. 2021;23:100334.

[19] Calvin CM, Batty GD, Der G, Brett CE, Taylor A, Pattie A, et al. Childhood intelligence in relation to major causes of death in 68 year follow-up: prospective population study. BMJ. 2017;357:j2708. 28659274 10.1136/bmj.j2708 PMC5485432

[20] Ludvigsson JF, Berglind D, Sundquist K, Sundström J, Tynelius P, Neovius M. The Swedish military conscription register: opportunities for its use in medical research. Eur J Epidemiol. 2022;37:767–77. 35810240 10.1007/s10654-022-00887-0 PMC9329412

[21] Bergström G, Berglund G, Blomberg A, Brandberg J, Engström G, Engvall J, et al. The Swedish CArdioPulmonary BioImage Study: Objectives and design. J Intern Med. 2015;278:645–59. 26096600 10.1111/joim.12384 PMC4744991

[22] Ekblom-Bak E, Börjesson M, Bergman F, Bergström G, Dahlin-Almevall A, Drake I, et al. Accelerometer derived physical activity patterns in 27.890 middle-aged adults: The SCAPIS cohort study. Scand J Med Sci Sports. 2022;32:866–80. 35080270 10.1111/sms.14131 PMC9302631

[23] Christensen SE, Möller E, Bonn SE, Ploner A, Wright A, Sjölander A, et al. Two new meal- and web-based interactive food frequency questionnaires: Validation of energy and macronutrient intake. J Med Internet Res. 2013;15:e109. 23739995 10.2196/jmir.2458 PMC3713929

[24] Nybacka S, Bertéus Forslund H, Wirfält E, Larsson I, Ericson U, Warensjö Lemming E, et al. Comparison of a web-based food record tool and a food-frequency questionnaire and objective validation using the doubly labelled water technique in a Swedish middle-aged population. J Nutr Sci. 2016;5:e39. 27752306 10.1017/jns.2016.29 PMC5048186

[25] Cerwinske LA, Rasmussen HE, Lipson S, Volgman AS, Tangney CC. Evaluation of a dietary screener: the Mediterranean Eating Pattern for Americans tool. J Hum Nutr Diet. 2017;30:596–603. 28168764 10.1111/jhn.12451

[26] Raff GL, Abidov A, Achenbach S, Berman DS, Boxt LM, Budoff MJ, et al. SCCT guidelines for the interpretation and reporting of coronary computed tomographic angiography. J Cardiovasc Comput Tomogr. 2009;3:122–36. 19272853 10.1016/j.jcct.2009.01.001

[27] Agatston AS, Janowitz WR, Hildner FJ, Zusmer NR, Viamonte M, Detrano R. Quantification of coronary artery calcium using ultrafast computed tomography. J Am Coll Cardiol. 1990;15:827–32. 2407762 10.1016/0735-1097(90)90282-t

[28] Touboul PJ, Hennerici MG, Meairs S, Adams H, Amarenco P, Bornstein N, et al. Mannheim carotid intima-media thickness consensus (2004–2006). An update on behalf of the Advisory Board of the 3rd and 4th Watching the Risk Symposium, 13th and 15th European Stroke Conferences, Mannheim, Germany, 2004, and Brussels, Belgium, 2006. Cerebrovasc Dis. 2007;23:75–80. 17108679 10.1159/000097034

[29] Sasaki JE, John D, Freedson PS. Validation and comparison of ActiGraph activity monitors. J Sci Med Sport. 2011;14:411–6. 21616714 10.1016/j.jsams.2011.04.003

[30] D’Orazio P, Burnett RW, Fogh-Andersen N, Jacobs E, Kuwa K, Külpmann WR, et al. Approved IFCC recommendation on reporting results for blood glucose. Clin Chem Lab Med. 2006;44:1486–90. 17163827 10.1515/CCLM.2006.275

[31] Herraiz-Adillo Á, Higueras-Fresnillo S, Ahlqvist VH, Berglind D, Syrjälä MB, Daka B, et al. Life’s Essential 8 and Life’s Simple 7 in Relation to Coronary Atherosclerosis: Results From the Population-Based SCAPIS Project. Mayo Clin Proc. 2024;99:69–80. 37843486 10.1016/j.mayocp.2023.03.023

[32] Carlstedt B. Cognitive abilities-aspects of structure, process and measurement. Dissertation. Gothenburg: University of Gothenburg; 2000.

[33] Carlstedt B, Mårdberg B. Swedish enlistment battery (SEB): Construct validity and latent variable estimation of cognitive abilities by the CAT-SEB. Int J Sel Assess. 1998;6:107–14.

[34] Carlstedt B, Gustafsson JE. Construct validation of the Swedish Scholastic Aptitude Test by means of the Swedish Enlistment Battery. Scand J Psychol. 2005;46:31–42. 15660631 10.1111/j.1467-9450.2005.00432.x

[35] Cho I, Chang HJ, Sung JM, Pencina MJ, Lin FY, Dunning AM, et al. Coronary computed tomographic angiography and risk of all-cause mortality and nonfatal myocardial infarction in subjects without chest pain syndrome from the CONFIRM registry (Coronary CT angiography evaluation for clinical outcomes: An international Multicenter Registry). Circulation. 2012;126:304–13. 22685117 10.1161/CIRCULATIONAHA.111.081380

[36] Lloyd-Jones DM, Allen NB, Anderson CAM, Black T, Brewer LC, Foraker RE, et al. Life’s Essential 8: Updating and Enhancing the American Heart Association’s Construct of Cardiovascular Health: A Presidential Advisory from the American Heart Association. Circulation. 2022;146:E18–43. 35766027 10.1161/CIR.0000000000001078 PMC10503546

[37] Laura M-G, Antonio A-C, Felipe JL, Samuel M-C, Gallardo L, Jorge G-U. Lower Body Muscular Strength as a Predictor of Health Indicators in Youth Population: A Systematic Review and Meta-Analysis. Sports Med Health Sci. 2025;8:273–90. 42125183 10.1016/j.smhs.2025.06.004 PMC13159449

[38] Harrell FE. Regression Modeling Strategies. 2nd ed. New York: Springer; 2015.

[39] Trahan LH, Stuebing KK, Fletcher JM, Hiscock M. The Flynn effect: a meta-analysis. Psychol Bull. 2014;140:1332–60. 24979188 10.1037/a0037173 PMC4152423

[40] Herraiz-Adillo Á, Eriksson H, Ahlqvist VH, Ballin M, Wennberg P, Daka B, et al. Blood Pressure in Adolescence and Atherosclerosis in Middle Age. JAMA Cardiol. 2026;11:14–24. 41259058 10.1001/jamacardio.2025.4271 PMC12631567

[41] Gale CR, Deary IJ, Batty GD, David Batty G. Intelligence and carotid atherosclerosis in older people: cross-sectional study. J Am Geriatr Soc. 2008;56:769–71. 18380686 10.1111/j.1532-5415.2008.01616.x

[42] Towfighi A, Zheng L, Ovbiagele B. Sex-specific trends in midlife coronary heart disease risk and prevalence. Arch Intern Med. 2009;169:1762–6. 19858433 10.1001/archinternmed.2009.318

[43] Bergström G, Persson M, Adiels M, Björnson E, Bonander C, Ahlström H, et al. Prevalence of Subclinical Coronary Artery Atherosclerosis in the General Population. Circulation. 2021;144:916–29. 34543072 10.1161/CIRCULATIONAHA.121.055340 PMC8448414

[44] Herraiz-Adillo Á, Ahlqvist VH, Higueras-Fresnillo S, Hedman K, Hagström E, Smidt MF, De, et al. Physical fitness in male adolescents and atherosclerosis in middle age: a population-based cohort study. Br J Sports Med. 2024;58:411–20. 38355280 10.1136/bjsports-2023-107663 PMC11041617

